# Bone Marrow Regulatory T Cells Are a Unique Population, Supported by Niche-Specific Cytokines and Plasmacytoid Dendritic Cells, and Required for Chronic Graft-Versus-Host Disease Control

**DOI:** 10.3389/fcell.2021.737880

**Published:** 2021-09-22

**Authors:** Jemma Nicholls, Benjamin Cao, Laetitia Le Texier, Laura Yan Xiong, Christopher R. Hunter, Genesis Llanes, Ethan G. Aguliar, Wayne A. Schroder, Simon Phipps, Jason P. Lynch, Huimin Cao, Shen Y. Heazlewood, Brenda Williams, Andrew D. Clouston, Christian M. Nefzger, Jose M. Polo, Susan K. Nilsson, Bruce R. Blazar, Kelli P. A. MacDonald

**Affiliations:** ^1^Division of Blood and Marrow Transplant and Cellular Therapies, Department of Pediatrics, Masonic Cancer Center, University of Minnesota, Minneapolis, MN, United States; ^2^Biomedical Manufacturing Commonwealth Scientific and Industrial Research Organization, Melbourne, VIC, Australia; ^3^Australian Regenerative Medicine Institute, Monash University, Melbourne, VIC, Australia; ^4^Immunology Department, QIMR Berghofer Medical Research Institute, Brisbane, QLD, Australia; ^5^Envoi Specialist Pathologists, Brisbane, QLD, Australia; ^6^Institute for Molecular Bioscience, The University of Queensland, Brisbane, QLD, Australia; ^7^Monash Biomedicine Discovery Institute, Monash University, Melbourne, VIC, Australia; ^8^Department of Anatomy and Developmental Biology, Monash University, Melbourne, VIC, Australia

**Keywords:** regulatory T cells, bone marrow, stem-cell transplantation, GVHD, FoxP3, TIGIT

## Abstract

Regulatory T cell (Treg) reconstitution is essential for reestablishing tolerance and maintaining homeostasis following stem-cell transplantation. We previously reported that bone marrow (BM) is highly enriched in autophagy-dependent Treg and autophagy disruption leads to a significant Treg loss, particularly BM-Treg. To correct the known Treg deficiency observed in chronic graft-versus-host disease (cGVHD) patients, low dose IL-2 infusion has been administered, substantially increasing peripheral Treg (pTreg) numbers. However, as clinical responses were only seen in ∼50% of patients, we postulated that pTreg augmentation was more robust than for BM-Treg. We show that BM-Treg and pTreg have distinct characteristics, indicated by differential transcriptome expression for chemokine receptors, transcription factors, cell cycle control of replication and genes linked to Treg function. Further, BM-Treg were more quiescent, expressed lower FoxP3, were highly enriched for co-inhibitory markers and more profoundly depleted than splenic Treg in cGVHD mice. *In vivo* our data are consistent with the BM and not splenic microenvironment is, at least in part, driving this BM-Treg signature, as adoptively transferred splenic Treg that entered the BM niche acquired a BM-Treg phenotype. Analyses identified upregulated expression of IL-9R, IL-33R, and IL-7R in BM-Treg. Administration of the T cell produced cytokine IL-2 was required by splenic Treg expansion but had no impact on BM-Treg, whereas the converse was true for IL-9 administration. Plasmacytoid dendritic cells (pDCs) within the BM also may contribute to BM-Treg maintenance. Using pDC-specific BDCA2-DTR mice in which diptheria toxin administration results in global pDC depletion, we demonstrate that pDC depletion hampers BM, but not splenic, Treg homeostasis. Together, these data provide evidence that BM-Treg and splenic Treg are phenotypically and functionally distinct and influenced by niche-specific mediators that selectively support their respective Treg populations. The unique properties of BM-Treg should be considered for new therapies to reconstitute Treg and reestablish tolerance following SCT.

## Introduction

Regulatory T cells (Treg) are an immunosuppressive CD4^+^ T cell subset essential for immune regulation and maintaining self-tolerance. Graft-versus-host disease (GVHD) is a tissue-destructive process of immune dysfunction in patients following allogeneic stem cell transplantation (SCT) (Taylor et al., 2002; Rezvani et al., 2006; Robb et al., 2012; Zhang et al., 2013; Le Texier et al., 2017). Preclinical and clinical studies documented a significant defect in peripheral Treg (pTreg) populations during GVHD (Chen et al., 2007; Beres and Drobyski, 2013; Le Texier et al., 2016), with an inverse correlation between donor graft Treg frequency and GVHD incidence and severity (Taylor et al., 2002; Rezvani et al., 2006; Robb et al., 2012; Zhang et al., 2013; Le Texier et al., 2017). Coupled with a Treg deficiency, the capacity of Treg to effectively suppress pathogenic T cells in GVHD has led to Treg cellular-based therapy for GVHD control (Hoffmann et al., 2002; Taylor et al., 2002; Edinger et al., 2003).

Previous studies demonstrated that Treg adoptive transfer can successfully attenuate GVHD and promote allogeneic hematopoietic stem cells (HSC) engraftment post-transplant (Hoffmann et al., 2002; Taylor et al., 2002; Rezvani et al., 2006; Wolf et al., 2007; Brunstein et al., 2011; Di Ianni et al., 2011; Koyama et al., 2016; Le Texier et al., 2016; McDonald-Hyman et al., 2016). As several major limitations impede the more widespread translation of Treg-based therapies (Brunstein et al., 2011; Hippen et al., 2011; Zhang et al., 2013; McDonald-Hyman et al., 2015; Koyama et al., 2016; Le Texier et al., 2016), clinical approaches have focused on increasing *in vivo* Treg number to promote immune tolerance flowing allogeneic SCT. Toward that end, recombinant interleukin-2 (IL-2) has been administered to drive *in vivo* Treg expansion after SCT. Recent clinical data has demonstrated that low dose IL-2 infusions can substantially augment pTreg frequency and reduce disease severity in in chronic graft-versus-host disease (cGVHD) patients (Zorn et al., 2005; Matsuoka et al., 2013; Koreth et al., 2016). However, only ∼50% of SCT patients benefit from IL-2 infusions (Curtis and Pavletic, 2016; Whangbo et al., 2019). Moreover, as IL-2 binds the heterotrimer IL-2Ra,b,g, such therapy may activate and expand conventional T cells (Tcon) (Pérol et al., 2014; Kim et al., 2016) with the potential to exacerbate inflammation.

Regulatory T cell are a phenotypically diverse and heterogenous cell population that can be categorized into sub-populations based on phenotypical, functional, and transcriptional signatures (Burzyn et al., 2013a; Richards et al., 2015). These Treg sub-populations are dynamic and readily influenced by diverse environmental mediators affecting the activation and differentiation of the distinct Treg cell subsets. There is mounting evidence that Treg populations exhibit unique tissue niche-specific phenotypic features that differentially contribute the regulation of local immune homeostasis and in a wide-range of human disease (Zaiss et al., 2010; Burzyn et al., 2013a, b; Chen et al., 2013; Richards et al., 2015; Zhou et al., 2015). Studies in mouse and man have shown that the BM is a natural reservoir of Treg *in vivo*. In contrast to other pTreg sources, BM-Treg represent between 20 and 60% of the CD4^+^ T cells situated within the BM compartment (Zou et al., 2004; Zhao et al., 2012a; Pierini et al., 2017; Zaretsky et al., 2017; Hirata et al., 2018; Camacho et al., 2020). There are burgeoning data and a developing recognition that Treg residing within the BM niche (BM-Treg) exhibit characteristics distinct from other pTreg populations (Zou et al., 2004; Zhao et al., 2012b; Le Texier et al., 2016; Fischer et al., 2019; Camacho et al., 2020).

Recent SCT studies provide evidence in support of BM-Treg as directly contributing to maintenance of the BM tissue and central for HSC homeostasis, engraftment, and survival in an immune privileged site within the BM niche (Fujisaki et al., 2011; Hirata et al., 2018). Niche residence protects HSCs from oxidative stress by regulating their quiescence within the BM (Hirata et al., 2018, 2019); reciprocally, HSC protects the BM niche from injury post-irradiation (Kakiuchi et al., 2021). Thus, the BM niche is likely much more than a reservoir for memory cells including Treg. Together these studies highlight the key, central, and diverse role of BM-Treg in establishing and maintaining BM homeostasis following SCT.

Current prophylactic immunosuppressive strategies to prevent cGVHD, namely the calcineurin inhibitor cyclosporin, may also impair BM-Treg reconstitution following SCT (Sugiyama et al., 2014). To reestablish longstanding immune tolerance, new approaches to effectively control cGVHD pathology and simultaneously support BM-Treg populations following SCT are needed. However, many of the mechanisms that locally influence BM-Treg are poorly defined. To develop more effective strategies that maximize cGVHD control in the clinical setting, a better understanding of mechanisms that locally influence BM-Treg stability, expansion, survival, and function is required. Here, we demonstrate that the BM niche represents a rich source of resting BM-Treg following SCT, that are uniquely regulated by the local tissue microenvironment and required for cGVHD control. We provide evidence that BM-Treg are phenotypically distinct from pTreg and have differential requirements for BM niche cellular and soluble mediators necessary for their reconstitution, maintenance, and function *in vivo*.

## Materials and Methods

### Animals

Inbred female C57BL/6, B6.SJL-*Ptprc^*a*^Peprc^*b*^* (*PTPrc*^a^, CD45.1^+^) and B6D2F1 mice were purchased from the Animal Resource Centre (Canning Vale, WA, Australia). B6.Foxp3.DTR (Stranges et al., 2007), B6.FoxP3-GFP (Fontenot et al., 2003), RAG1^–/–^ and B6.BDCA2-DTR (Lynch et al., 2018) mice were bred and maintained in-house at QIMR Berghofer Medical Research Institute. B6.IL-10-GFP, B6.FoxP3-RFP were originally provided by Alexander Rudensky (Memorial Sloan Kettering Cancer Institute), and B6.*FoxP3-RFP*x*IL10-GFP* mice (Edwards et al., under review) were intercrossed and kindly provided by Christian Engwerda (QIMR Berghofer Medical Research Institute). *Atg7^*fl/fl*^* (Kuma et al., 2004) mice were provided by Masaaki Komatsu (Tokyo Metropolitan Institute of Medical Science). *FoxP3*^*Cre–IRES–YFP*^ mice (Rubtsov et al., 2008) were bred with *Atg7*^*fl/fl*^ to generate *Atg7^*fl/fl*^-FoxP3cre^+^* mice. Female mice were used between 8 and 12 weeks, unless otherwise indicated. All mice were maintained in-house under pathogen-free conditions. All animal procedures were conducted with the approval of the QIMR Animal Ethics Committee under the animal ethics number A1503-603M and in accordance with the Australian Code of Practice for the Care and Use of Animals for Scientific Purposes (National Health and Medical Research Council, Canberra, Australia).

### Stem Cell Transplantation

On day 0, recipient mice received 1,100 cGy (B6D2F1) or 1,000 cGy (C57BL/6, B6) total body irradiation split into two doses separated by 3 h. T cell depleted bone marrow (TCD-BM) and splenic T cells were purified using magnetic bead depletion (purity > 85%) as described (Burman et al., 2007). Recipients were transplanted with 5 × 10^6^ (B6) TCD-BM from C57BL/6, B6.*FoxP3-GFP.DTR*, B*6.WT.FoxP3cre-YFP^+^*, B*6.Atg7^*fl/fl*^*. *FoxP3cre-YFP^+^*, or BDCA2-DTR donor mice as indicated, with or without splenic T cells from B6.*FoxP3-GFP*, B6.*FoxP3-RFP*, or C57BL/6 donors. Transplanted mice were monitored daily to evaluate clinical GVHD scores as previously published (Hill et al., 1997). Briefly, weight loss, posture, activity, fur texture, and skin integrity were measured. Mice with a score greater than or equal to 6 were culled, and the date of the following day is recorded as the death date. Where indicated, recipient mice received intraperitoneal injections IL-2 (0.5 μg)/JES6-1 anti-IL-2 mAb (25 μg) complexes on days 21–34. In studies where Treg or plasmacytoid dendritic cells (pDC) were selectively depleted from BM grafts during acute GVHD, mice were injected intraperitoneally with diptheria toxin (DT) (1 μg/animal, in 200 μl total volume) twice weekly from day 21 post-transplant, as indicated.

For adoptive transfer experiments, on day 0, B6.RAG1^–/–^ recipient mice received 0.5 × 10^6^ sort purified B6.FoxP3.GFP^+^ Treg isolated from the spleens of B6.FoxP3.GFP mice. BM and SP were harvested from recipient mice 4 weeks following adoptive transfer for flow cytometry analysis.

### *In vivo* Cytokine Administration in Naïve Mice

To assess T cell responses to *in vivo* cytokine stimulation B6.FoxP3.GFP mice were administered with either saline or recombinant murine cytokines over 4 days. Control animals received saline intraperitoneal injections daily for 4 days. IL-2 complex was administered daily for 4 days: 0.5 μg recombinant mouse IL-2 (eBioscience^TM^) with 25 μg anti-IL-2 (BioXcell). IL-15 complex was administered on days 1 and 3: 7 μg IL-15Rα-Fc (R&D) with 0.75 μg of recombinant mouse IL-15 (eBioscience^TM^). IL-33 was administered daily for 4 days: 1 μg of recombinant mouse IL-33 (Peprotech). IL-9 daily for 4 days: 50 ng of recombinant mouse IL-9 (*In Vitro* Technologies TY Ltd.). IL-7 complex daily for 4 days: 1 μg of recombinant mouse IL-7 with 5 μg of anti-IL-7 (Jomar Life Research). The absolute number of CD4^+^FoxP3^+^ Treg and CD4^+^FoxP3^neg^ Tcon in either the spleen and BM of control and treated mice was then assessed on day 6 following the cytokine treatment regime.

### Flow Cytometric Analysis

Single-cell suspensions of spleen and bone marrow cells were stained with fluorochrome- or biotin-conjugated antibodies against antigens as listed in Table 1. Intracellular staining for Foxp3 and MCL-1 was performed after fixation and permeabilization using the eBiosciences Foxp3 staining kit. Sample data were acquired on a LSRFortessa flow cytometer (BD Biosciences) and analyzed using FlowJo software (TreeStar) version 10. Cell sorting was performed using an ARIA III Cell Sorter (BD Biosciences).

**TABLE 1:** Antibodies used for surface and intracellular flow cytometry analysis.

Functional/Secondary Marker	Antibody information
AnnexinV	**PE;** BD Pharmingen
CCR5	**Biotin;** HM-CCR5, Biolegend
CD101 (Igsf2)	**AF647;** clone 307707, BD Pharmingen
CD103	**Pacific Blue;** clone 2E7, Biolegend
CD11c	**PE;** clone N418, Biolegend
	**APC;** clone N418 Catalog #117310 Biolegend
CD127 (IL-7Rα)	**BV421;** clone A7R34, Biolegend
CD193 (CCR3)	**PerCP-Cy5.5;** clone J073E5, Biolegend
CD215 (IL-15Rα)	**PE;** 6B4C88, Biolegend
CD25	**APC;** clone PC61, Biolegend
	**BV605;** PC61, Biolegend
CD3	**APC-Cyanine7;** clone 17A2, Biolegend
CD304	**PerCP-eFluor 710;** clone 3DS304M, eBioscience
CD317	**PerCP-eFluor 710;** clone eBio927 Catalog #46-3172-82 eBioscience
CD326 (EpCam)	**BV605;** clone G8.8, Biolegend
	**PE-Cyanine7;** G8.8, Biolegend
CD39	**PE-Cyanine7;** clone Duha59, Biolegend
CD4	**Pacific Blue;** RM4.5, Biolegend
	**BV605;** clone RM4.5, Biolegend
	**Biotin;** clone H129.9, BD Biosciences
	**APC;** clone GK1.5, Biolegend
CD49b	**APC;** Hmα2, Biolegend
CD62L	**PerCP-Cy5.5;** clone MEL-14, Biolegend
CD69	**Pacific Blue;** clone H1.2F3, Biolegend
	**Pacific Blue;** H1.2F3, Biolegend
CD73	**PerCP-Cy5.5;** clone TY/11.8, Biolegend
FoxP3	**AF647;** clone 150D, Biolegend
GARP	**PerCP-eFluor 710;** clone YG1C86, eBioscience
	**PE;** clone YG1C86, eBioscience
IL-33Rα (IL1RL1, ST2)	**PE;** clone DIH9, Biolegend
IFN-γR	**Biotin;** clone 2E2 Biolegend
Ki67	**BV786;** clone B56, BD Biosciences
	**PE-Cyanine7;** clone 16A8, Biolegend
KLRG1	**PE-Cyanine7;** clone 2F1/KLRG1, Biolegend
MCL-1	**AF647;** clone 19c4-15 (produced in house at WEHI, and kindly provided by Daniel Gray)
MHCII	**FITC;** clone M5/114.15.2 Catalog # 107606 Biolegend
PD-1	**PE-Cyanine7;** clone RMP1-30, Biolegend
pSTAT5	**AF647;** clone pY694, BD Pharmingen
Sca-1	**PE-Cyanine7;** clone E13-161.7, Biolegend
	**PE-Cyanine7;** clone D7, Biolegend
Streptavidin	**BV605;** Biolegend
	**PE-Cyanine7**; Biolegend
	**AF700;** ThermoFisher
TCF-7/TCF-1	**PE;** clone S33-966, BD Pharmingen
TIGIT	**BV605;** clone 1G9, BD Bioscience
	**PE;** clone 1G9, Biolegend
	**APC;** 1G9, Biolegend

### *In vitro* Suppression Assay

For *in vitro* suppression assays, CFSE-labeled sort purified splenic CD4^+^ T cells from iVac-cre.BFP^+^ mice, purified using previously described magnetic bead depletion, were seeded at 1 × 10^4^ cells/well in 96-well round-bottom plates, with DCs (B6) at 1,000 cells/well, in the absence or presence of Tregs (B6.Foxp3-GFP) at 1,250, 2,500, or 5,000 cells/well (8:1, 4:1, or 2:1 Tcon:Treg, respectively), and supplemented with anti-CD3 (2C11, 1 μg/ml). Cells were harvested for CFSE dilution analysis at 72 h of culture.

### RNA Isolation, Library Preparation, and Sequencing

RNA was extracted with Qiagen’s RNeasy micro kit from >100,000 FACS isolated cells as per instructions. For generation of sequencing libraries, 15 ng of RNA (RIN value > 7) were submitted to SPIA amplification (NuGen). Four biological replicates per condition were sequenced using the HiSeq 3,000 sequencing platform (Illumina, San Diego, CA, United States). Each library was sequenced single-end with a 80 nucleotide read length. The targeted number of sequencing reads per sample was ∼30–50 million.

### Cell Cycle Analysis

Spleen and BM cells from B6.*FoxP3-GFP* mice were harvested and immunolabelled with surface antibody cocktail containing anti-CD3 and anti-CD4-biotin labeling antibodies. The cells were then stained for anti-Foxp3-AF647, anti-Ki67-BV786, and Hoechst 33342 (Invitrogen; H3570) using the eBioscience^TM^ Foxp3/Transcription Factor Staining kit (ThermoFisher) following the manufacturer’s instructions. Spleen and BM CD3^+^CD4^+^FoxP3^+^ Treg were analyzed by flow cytometry for Ki67 and Hoechst (Hö) staining, with different stages of cell cycle defined as G0 (Ki67^neg^Hö^low^), G1 (Ki67^+^Hö^low^), and S-M (Ki67^+^Hö^high^).

### Real-Time Quantitative PCR

Total RNA was extracted from sorted cells using the RNeasy Micro kit (QIAGEN), and reverse transcription was performed using SuperScript III Reverse Transcriptase (Life Technologies). RNA relative expression was determined using Taqman gene expression assays (Life Technologies). The housekeeping gene *HPRT* was used to normalize the gene expression to the starting quantity of RNA. PPARγ mRNA expression was calculated using the 2^–ΔΔCt^ method (Livak and Schmittgen, 2001).

### pSTAT5

CD4^+^ T cells were isolated from the spleen and BM of B6.FoxP3-GFP mice and incubated for 15 min at 37°C with or without recombinant murine IL-2 (100 ng/mL), IL-7 (50 ng/mL), IL-9 (20 ng/mL), or IL-33 (25 ng/mL). Cells were processed according to manufacturer’s protocol (BD Phosflow Perm Buffer III).

### Cytokine Quantification in BM and Spleen

The long bones (femur, tibia, and iliac crest) from C57BL/6 mice were cleaned of muscle and tissue and then flushed with cold PBS (250 μl) to isolate the BM cells and extracellular fluid. Spleens from C57BL/6 mice were harvested, mashed between two microscope slides until homogenized, placed in an Eppendorf vial and then cold PBS (200 μl) was added and the mixture vortexed vigorously. The cell suspensions from both bone marrow and spleen were centrifuged at 400 × g for 5 min and the supernatant was then transferred to a clean Eppendorf vial. The supernatant was centrifuged at 10,000 × g for 10 min and stored frozen at −80°C. The resultant cell pellets from BM and spleen were resuspended in PBS (2% FBS) and enumerated for subsequent data normalization. The supernatant samples were analyzed using the Quantibody^®^ assay at Crux Biolabs and protein concentration normalized relative to cell number.

### Statistics

Data are shown as mean ± SEM. Statistical significance was determined using an unpaired 2-tailed Mann-Whitney *U* test, Wilcoxon matched-paired test, log-rank (Mantel-Cox) test, or paired *t* test when appropriate (^∗^*P* < 0.05; ^∗∗^*P* < 0.01; ^∗∗∗^*P* < 0.001; ^****^P < 0.0001). Statistical analyses were performed using GraphPad Prism software version 6.01.

## Results

### Treg Are Highly Enriched Within the BM and Represent a Phenotypically Distinct Treg Population

**FIGURE 1:**
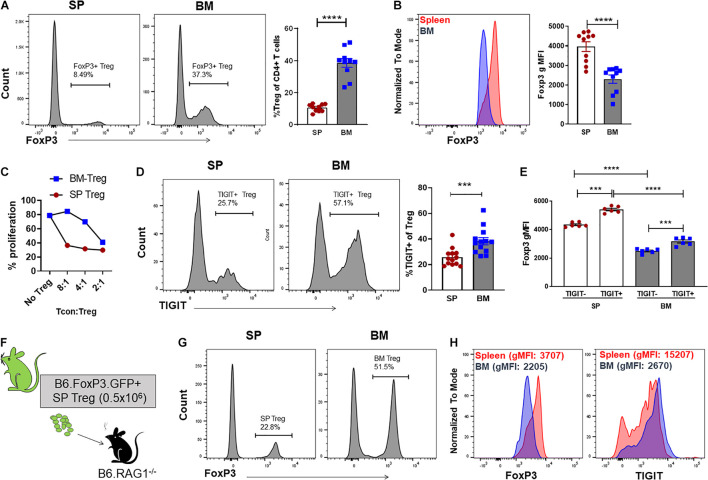
The BM is an enriched source of Treg which are phenotypically unique and directly influenced by the BM tissue niche. **(A,B)** Flow cytometry analysis of FoxP3 expression in CD4^+^ T cells isolated from naïve C57BL/6 mice aged 8–12 weeks (*n* = 10). **(A)** Representative histogram and frequency (%) of FoxP3^+^ expression in CD4^+^CD3^+^ SP and BM T cells **(B)** Representative histogram and gMFI of intracellular FoxP3 expression in CD4^+^CD3^+^ T cells isolated from the SP or BM. **(C)** *in vitro* analysis of the T cell suppressive capacity of FoxP3^+^ Treg isolated from either the SP or BM of B6.*FoxP3-GFP* transgenic mice (representative of independent experiments, *n* = 2). **(D)** Representative histogram and frequency (%) of TIGIT^+^ expression in CD4^+^CD3^+^ T cells from the BM or SP (*n* = 10). **(E)** gMFI of intracellular FoxP3 expression in TIGIT^neg^ and TIGIT^+^ CD4^+^CD3^+^ T cells isolated from the SP or BM (*n* = 6). **(G–H)** Cytometry analysis of FoxP3^+^ Treg populations 28 days following adoptive Treg transfer. B6.RAG^–/–^ recipients were administered with 0.5 × 10^6^ FoxP3^+^GFP^+^ Treg isolated from the SP of B6.FoxP3.GFP^+^ transgenic mice. **(F)** Outline of adoptive transfer transplant strategy. **(G)** Frequency (%) and FoxP3^+^ expression in CD4^+^CD3^+^ T cells from the BM and SP of recipient mice. **(H)** Representative histograms of intracellular FoxP3 expression and TIGIT^+^ expression in CD4^+^CD3^+^ T cells isolated from the SP or BM of recipient mice. Data are shown as mean ± SEM, and statistical significance was determined using paired *t* test (***P < 0.001; ****P < 0.0001). Statistical analyses were performed using GraphPad Prism version 6.01 software. Treg, regulatory T cell; SP, spleen; BM, bone marrow; gMFI, geometric mean of fluorescence intensity; TIGIT, T cell immunoreceptor with Ig and ITIM domains.

We have previously reported that the Treg population that resides in the BM is enriched with an autophagy-dependent TIGIT^+^ Treg population critical for establishing tolerance and controlling GVHD following SCT (Le Texier et al., 2016). Consistent with previous reports (Zou et al., 2004; Wei et al., 2006; Camacho et al., 2020), characterization of the BM resident Treg (BM-Treg) in naïve B6 mice aged 8–12 weeks demonstrated that Foxp3^+^ Treg are highly enriched in the CD4^+^ T cell compartment within the BM as compared to the spleen (SP) (BM: ∼40% vs SP: ∼10%). Further, BM-Treg expressed reduced FoxP3 levels compared to their splenic counterparts (Figures 1A,B). As FoxP3 is increased upon Treg activation and is critical for Treg suppressive function (Hori et al., 2003), reduced FoxP3 expression by BM-Treg suggests these Treg are in a less activated or functional state than pTreg (Liu et al., 2013; Rothstein and Camirand, 2015). Consistent with this, we show that compared to SP Treg, BM-Treg have a reduced capacity to suppress splenic Tcon proliferation *in vitro* (Figure 1C). However, the BM-Treg compartment is enriched in TIGIT-expressing Treg (BM: ∼36%, vs SP: ∼28%) which identifies a highly functional and activated memory Treg population (Figure 1D; Le Texier et al., 2016). While the TIGIT^+^ Treg within the BM and SP exhibited higher FoxP3 expression than TIGIT^neg^ Treg, FoxP3 expression was reduced in both TIGIT^+^ and TIGIT^neg^ Treg subsets in the BM as compared to the SP (Figure 1E). Comparing FoxP3 expression in CD4^+^ Treg across multiple lymphoid organs demonstrated that BM-Treg expressed the lowest FoxP3 levels irrespective of their TIGIT status (Supplementary Figure 1). To investigate this further, we adoptively transferred FoxP3^+^GFP^+^ Treg isolated from the SP of B6.FoxP3-GFP mice into B6.RAG1^–/–^ recipients (Figure 1F). Four weeks following adoptive transfer, we observed a marked enrichment of FoxP3^+^ Treg within the BM compartment of recipient mice (Figure 1G) and, despite originating from the SP, Treg isolated from the BM of recipient mice acquired a BM-Treg phenotype, including reduced expression of FoxP3 and enriched TIGIT expression (Figure 1H). Taken together, these data suggest that the BM microenvironment influences the phenotype and function of locally residing Treg and may play a central role in development and/or maintenance of the BM-Treg signature.

**FIGURE 2:**
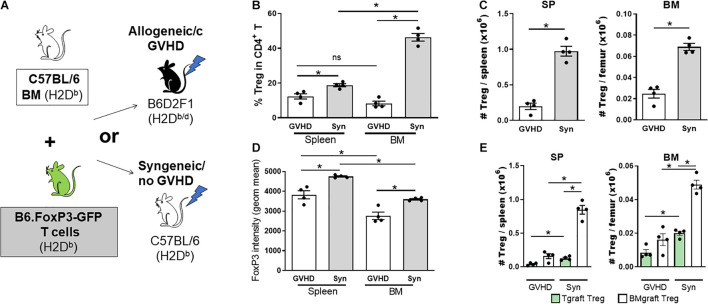
The BMgraft is a rich source of Treg following SCT, however, BMgraft derived Treg but fail to reconstitute within the both BM and SP during cGVHD. **(A–E)** Cytometry analysis of FoxP3^+^ Treg populations 28 days following SCT. Irradiated syngeneic C57BL/6 (H2D^b^) or allogeneic B6D2F1 (H2D^b/d^) recipients were transplant with BMgrafts from C57BL/6 mice supplemented with SP Tgrafts from B6.*FoxP3-GFP* transgenic mice (*n* = 4). **(A)** Outline of syngeneic and allogeneic transplant strategy. **(B)** Frequency (%) and **(C)** Total number of FoxP3^+^CD4^+^CD3^+^ T cells isolated from the BM and SP of transplanted mice. **(D)** gMFI of intracellular FoxP3^+^ expression in CD4^+^CD3^+^ T cells from the BM and SP of transplanted mice. **(E)** Total number of Tgraft derived (GFP^+^) or BMgraft derived (GFP^neg^) FoxP3^+^ CD4^+^CD3^+^ cells in the BM or SP of transplanted mice. Data are shown as mean ± SEM. Statistical significance was determined using an unpaired 2-tailed Mann-Whitney U test (*P < 0.05). Statistical analyses were performed using GraphPad Prism version 6.01 software. cGVHD, chronic graft versus host disease; SCT, stem cell transplantation.

A failure in Treg homeostasis, with an associated reduction in pTreg numbers, contributes to cGVHD pathogenesis in both patients and mice (Zorn et al., 2005; Koyama et al., 2016; McDonald-Hyman et al., 2016). To assess the impact of cGVHD on the BM Treg compartment, we transferred B6 BM (5 × 10^6^) supplemented with splenic T cells (0.5 × 10^6^) from B6.FoxP3-GFP donors into lethally irradiated allogeneic B6D2F1 or syngeneic B6 recipients (Figure 2A). Late post-transplant, the Treg pool can be comprised of Treg derived from either the mature T cells contained in the graft (Tgraft Treg) or those emerging from the BM compartment (BMgraft Treg). The relative impact on these Treg populations was assessed using B6.FoxP3-GFP T cells. On day 28 after transplant, intracellular FoxP3 staining used to quantify all CD4^+^ Treg revealed a significant reduction in Treg frequency within the CD4 T cell compartment of the SP and to an even greater extent in the BM of mice receiving GVHD-inducing grafts as compared to syngeneic graft recipients (Figure 2B). Notably, in syngeneic recipients, similar to that seen in naïve mice, there was an increased proportion of Treg in the BM compartment compared to the SP, however, Treg failed to enrich in the BM of GVHD recipients. Overall, there was a significant reduction in CD4^+^FoxP3^+^ Treg numbers and FoxP3 expression in SP and BM of GVHD mice with lower FoxP3 expression in BM than SP residing Treg (Figures 2C,D). In syngeneic recipients, the majority of Treg in both the SP (∼85%) and BM (∼70%) were derived from the BMgraft and not the Tgraft GFP^+^CD4 Treg (Figure 2E). While GVHD impaired the reconstitution of both Tgraft and BMgraft Treg in the SP and BM, the relative impact of GVHD was greatest on the BMgraft Treg numbers.

**FIGURE 3:**
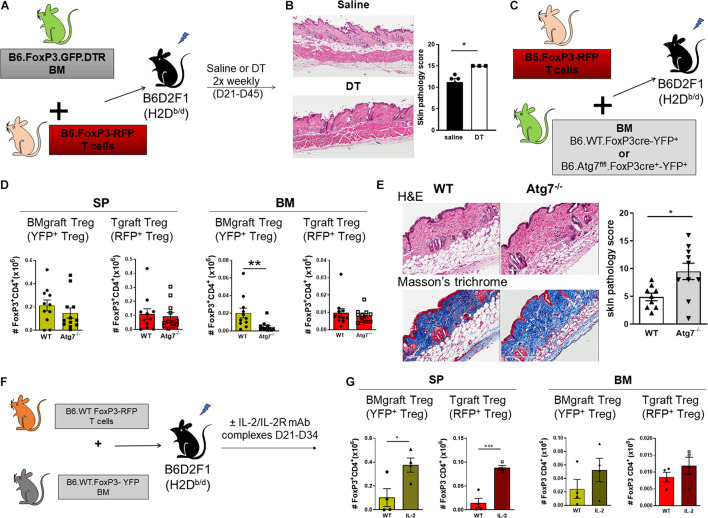
BMgraft Treg are required for cGVHD control. **(A,B)** Irradiated allogeneic B6D2F1 (H2D^b/d^) recipients were transplant with SP Tgrafts from B6.*FoxP3-RFP* transgenic mice and BMgrafts from B6.*FoxP3-GFP-DTR* transgenic mice. Transplanted mice were administered with either saline or DT twice weekly from day 21 until day 45 post-transplant (*n* = 3, 1 experiment). **(A)** Outline of allogeneic transplant strategy. **(B)** Representative images of H&E staining of the skin and pathology scoring from histopathology analysis of the skin of allogeneic transplant recipients. Original magnification, ×200. **(C–E)** Irradiated allogeneic B6D2F1 (H2D^b/d^) recipients were transplanted with SP Tgrafts from B6.*FoxP3-RFP* transgenic mice and BMgrafts from either Atg7fl/fl-FoxP3cre^+^YFP^+^ (Atg^7–/–^) or WT-FoxP3cre^+^YFP^+^ (WT) transgenic mice. **(C)** Outline of allogeneic transplant strategy. **(D)** Enumeration of BMgraft derived (YFP^+^) and Tgraft derived (RFP^+^) Treg in the SP and BM of WT (*n* = 11 from two independent experiments) or ATG^7–/–^ (*n* = 12 from two independent experiments) BMgraft recipients on day 35 after transplant. **(E)** Representative images of H&E staining and Masson’s trichrome staining of the skin and pathology scoring from histopathology analysis of the skin of WT (*n* = 9 from two independent experiments) or ATG^7–/–^ (*n* = 10 from two independent experiments) BMgraft recipients. **(F,G)** Irradiated allogeneic B6D2F1 (H2D^b/d^) recipients were transplant with SP Tgrafts from B6.*FoxP3-RFP* transgenic mice and BMgrafts from WT-FoxP3^+^YFP^+^ transgenic mice. Transplanted mice were administered with either saline or IL-2/IL-2R mAB complex twice weekly from day 21 to day 34 post-transplant (*n* = 4). **(F)** Outline of allogeneic transplant strategy. **(G)** Enumeration of BMgraft derived (YFP^+^) and Tgraft derived (RFP^+^) Treg in the SP and BM on day 35 post-transplant. Data are shown as mean ± SEM. Statistical significance was determined using an unpaired 2-tailed Mann-Whitney U test (*P < 0.05; **P < 0.01; ***P < 0.001). Statistical analyses were performed using GraphPad Prism version 6.01 software. Atg, autophagy-related gene; DT, diptheria toxin.

We next assessed the contribution of BMgraft Treg to the control of GVHD. Again using the B6 → B6D2F1 cGVHD model, which is characterized by the development of sclerodermatous cGVHD (Koyama et al., 2016), we transferred BM from B6.FoxP3.GFP.DTR donor mice in which diphtheria toxin receptor (DTR) is driven off the FoxP3 promoter to facilitate specific Treg depletion in diphtheria toxin (DT) treated mice. To restrict Treg depletion to the BMgraft Treg compartment, B6.FoxP3.GFP.DTR BM grafts were supplemented with splenic T cells from a B6.FoxP3-RFP donor that is unaffected by DT administration (Figure 3A). On day 50 post-transplant, recipients administered DT twice weekly from day 21, when thymic T cell reconstitution begins, exhibited exacerbated skin cGVHD compared to those receiving saline, confirming a functional role for BMgraft Treg in contributing to the control of cGVHD (Figure 3B).

Our earlier studies using BM and T grafts from Atg7fl/fl-FoxP3cre^+^ mice with disabled autophagy specifically in FoxP3^+^ Treg demonstrated that Treg intrinsic autophagy is required for efficient Treg reconstitution and GVHD control (Le Texier et al., 2016). To examine the requirement for autophagy in BMgraft Treg engraftment and GVHD control, we next utilized grafts comprised of BM from WT-FoxP3cre-YFP^+^ or Atg7^fl/fl^-FoxP3cre^+^-YFP^+^ donors supplemented with B6.FoxP3-RFP T cells (Figure 3C). As compared to WT-FoxP3cre-YFP^+^ BM recipients, enumeration of BMgraft and Tgraft Treg in SP and BM on D35 after transplant revealed that selective Treg autophagy deficiency significantly reduced YFP^+^BMgraft Treg numbers in BM but not SP. Irrespective of the BM source, RFP^+^Tgraft Treg numbers were similar in both the SP and BM compartments (Figure 3D). Importantly, this Treg perturbation, which was restricted to the BM compartment, was associated with increased skin cGVHD pathology including increased dermal thickening and collagen deposition in the skin of recipients of autophagy-deficient BM grafts (Figure 3E).

We have previously shown the administration of anti–IL-2 mAb/IL-2 complexes expands pTreg and reduces cGVHD severity (McDonald-Hyman et al., 2016). Whether anti-IL-2mAb/IL-2 complexes improve BM-Treg numbers during cGVHD is unknown. To address this issue, we performed transplants where B6D2F1 recipients received grafts comprised of BM from FoxP3-YFP donors supplemented with T cells from B6.FoxP3-RFP mice that permits discrimination of Tgraft-derived from BM-derived Treg post-transplant (Figure 3F). Anti–IL-2 mAb/IL-2 complexes effectively expanded SP-Treg, including both Tgraft and BMgraft-derived Treg (Figure 3G). In contrast, there was minimal impact on the BM-Treg, suggesting that additional Treg enhancing therapeutic strategies are required to specifically improve BM-Treg numbers for better control of cGVHD.

### BM-Treg Exhibit a Distinct Transcriptional Profile

**FIGURE 4:**
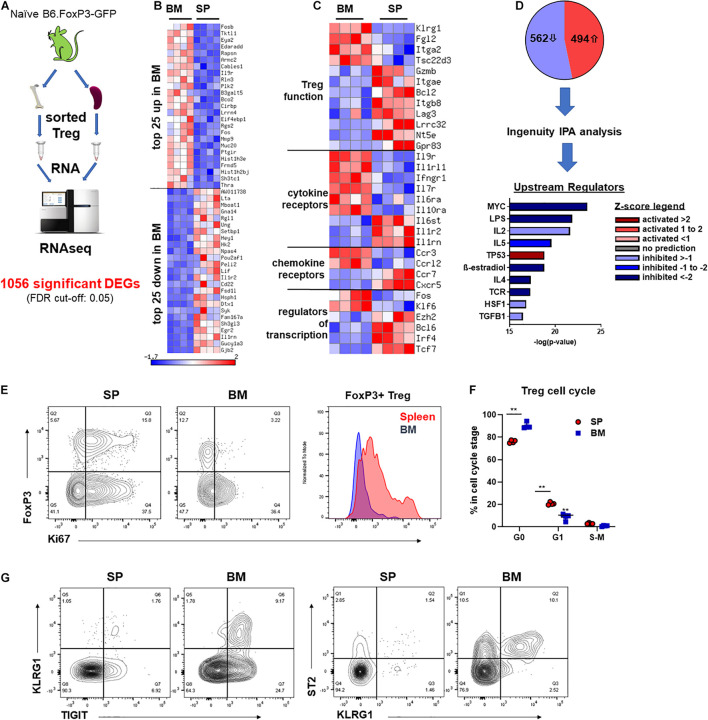
BM-Treg exhibit distinct transcriptional and phenotypic signatures. **(A–D)** RNAseq analysis of FoxP3^+^ Treg sort purified from either the BM or SP of 12-week-old naïve B6.FoxP3-GFP mice. **(A)** Outline of RNAseq analysis strategy. **(B)** Heatmap showing bulk RNRseq analysis of DEGs in FoxP3^+^ cells (*n* = 3 replicated per tissue). Top 25 upregulated and downregulated DEGs in BM-Treg. **(C)** Heatmap showing curated analysis of DEGs in BM-Treg. Differential expression of genes associated with Treg activation and function. **(D)** Ingenuity IPA of likely upstream regulators. **(E)** Representative flow cytometry analysis of Ki67 expression relative to FoxP3^+^ expression in CD4^+^CD3^+^ T cells isolated from the SP or BM. Representative histogram of Ki67 expression FoxP3^+^CD4^+^ Treg from the SP or BM. **(F)** Cell cycle analysis of FoxP3^+^ Treg isolated from either the BM or SP of naïve B6.FoxP3-GFP mice (*n* = 3). **(G)** Flow cytometry analysis of TIGIT^+^, KLRG1^+^ and ST2^+^ Treg in the SP and BM. RNAseq, RNA sequencing; DEGs, differentially expressed genes; IPA, interpretative phenomenological analysis. Data are shown as mean ± SEM. Statistical significance was determined using an unpaired 2-tailed Mann-Whitney U test (**P < 0.01).

To gain a better understanding of the phenotypic and functional differences between Treg of BM and splenic origin, we preformed bulk RNA sequencing (RNAseq) analysis on FoxP3^+^ Treg isolated from the BM or SP of naïve B6.FoxP3-GFP mice (Figure 4A). We identified 1056 differentially expressed genes (DEGs; 526 downregulated and 494 upregulated) in BM-Treg confirming that BM-Treg are transcriptionally distinct from SP Treg (Figure 4B). In line with recent studies, we observed increased expression of several genes [KLRG1, Fgl2, Itga2 (CD49b), and Tsc22d3 (GILZ)] that were consistent with an enrichment of an activated memory Treg phenotype within the BM (Figure 4C; Joller et al., 2014; Camacho et al., 2020). In contrast, and in concordance with our observed reduction in suppressive function in BM-Treg, multiple genes associated with Treg function [Gzmb, Itgea (CD103), Lag3, Lrrc32 (GARP), Nt5e (CD73), and Gpr83] were downregulated in BM-Teg. Relative to SP-Treg, BM-Treg exhibited divergent patterns of cytokine and chemokine receptor gene expression with BM-Treg expressing elevated levels of cytokine receptors for IL-9, IL-33, IFNγ, IL-7, IL-6, IL-10, and chemokine receptors CCR3 and CCRL2. In BM resident Treg, differences in transcription factor expression including the downregulation of FoxP3 interacting transcription factors Ezh2, Irf4, and Tcf7 were noted. Thus, the BM-Treg exhibit a unique transcriptional profile with differential expression of genes associated with Treg function, activation, cytokine responsiveness, and migration.

### Transcriptional Analysis Shows BM-Treg Are in a Less Active, More Quiescent State

Using Ingenuity interpretative phenomenological analysis (IPA) of likely upstream regulators (USRs) we observed a signature in BM-Treg consistent with a loss of activity and gain of a quiescent state. MYC was one of the most significantly dysregulated USRs predicated by IPA (Figure 4D). BM-Treg MYC activity was predicted to be significantly downregulated, with a z-score of −5.39. Additionally, 127 of the 1053 DEGs identified by our bulk RNAseq analysis are direct MYC targets, 78 of which were differential expressed in BM-Treg consistent with inhibition of MYC signaling. MYC drives cell cycle and proliferation in response to TCR and IL-2 (Tzachanis et al., 2004; Chapman et al., 2020; Rodriguez et al., 2021) which are also predicted to be downregulated in BM-Treg (Figure 4D) suggesting this subset is in a more quiescent and less proliferative state than pTreg. Supporting this, fewer BM-Treg expressed Ki-67, and cell cycle analysis showed an increased proportion of BM-Treg over SP-Treg in G0, and thus quiescent (Figures 4E,F). Together these data demonstrate that while BM-Treg may be enriched with activated memory Treg subsets, they are simultaneously in a more resting and quiescent state than pTreg.

Extending our phenotypic analysis as guided by the DEG list and literature, we performed flow cytometric profiling to identify unique features of BM-resident Treg. Analysis of a broad panel of functional markers on CD4^+^FoxP3^neg^ Tcon and CD4^+^FoxP3^+^ Treg from the SP and BM of 12 week old WT B6 mice identified several markers that were specifically increased on BM-Treg compared to SP-Treg (Supplementary Figure 2). Another set of markers (CD69, CD49b, and CD39) were observed to be increased on both Tcon and Treg in the BM, while both GARP and TCF-1 (encoded by Tcf7) were downregulated on BM-Treg (Supplementary Figure 2). Differential expression of functional markers was confirmed using B6.FoxP3-GFP reporter mice (Supplementary Figure 3), and the relative expression of these antigens was further assessed in TIGIT^+^ and TIGIT^neg^ CD4^+^FoxP3-GFP^+^ Treg sub-populations. As noted for TIGIT expression, the majority of these Treg markers [PD-1, CD103, EpCam (CD326), CD39, CD103, and CD69] were expressed on a BM-Treg subset, rather than globally upregulated. Sca-1 expression was noted to be specifically upregulated within the TIGIT^+^ Treg subset of BM-Treg compared to TIGIT^+^ SP-Treg. In contrast, CD127 (IL-7R), CD73, and INF-γR expression were globally upregulated on BM-Treg population. Thus, through recruitment and/or the local influence of the microenvironment, the BM harbors distinct and heterogeneous Treg subsets.

To further assess the heterogeneity of BM-Treg we surveyed key BM-Treg signature marker expression as related to TIGIT. KLRG1 marked a distinct population of TIGIT^+^ Treg that co-expressed ST2, signature markers of non-lymphoid tissue Treg (Figure 4G). Using gene set enrichment analysis of the RNAseq data, we identified an enrichment of genes related to visceral adipose tissue (VAT) Treg (Feuerer et al., 2009), and PPARγ signaling in Treg which drives VAT Treg accumulation, phenotype and function (Cipolletta et al., 2012; Supplementary Figure 4A). IPA identified PPARγ signaling as a top upregulated canonical pathway in BM-Treg (Supplementary Figure 4B). Although the level of PPARγ did not pass gene expression filters during RNAseq analysis, as GSEA and IPA analysis suggested PPARγ to be active in the BM-Treg, we performed RT-qPCR and confirmed upregulation of PPARγ in BM-Treg relative to SP-Treg (Supplementary Figure 4C), indicative of a VAT-like KLRG1^+^ST2^+^ tissue Treg subset enrichment in BM.

### BM-Treg Represent a Highly Enriched and Heterogeneous Population From an Early Age

**FIGURE 5:**
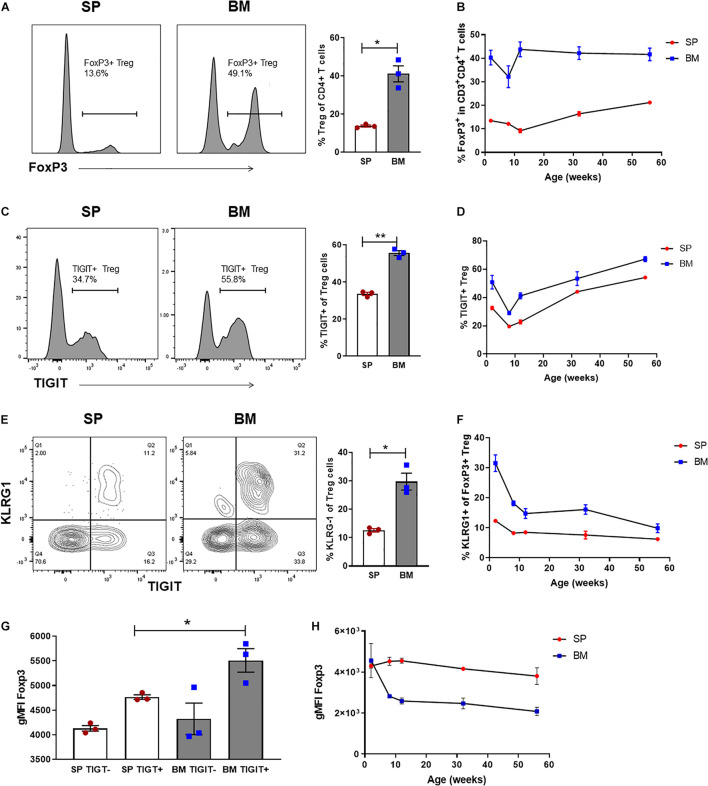
BM-Treg represent a heterogenous and phenotypically distinct population from an early age. Flow cytometry analysis CD4^+^ T cells isolated from naïve C57BL/6 mice aged 2–52 weeks (*n* = 3). **(A)** Representative histogram and frequency (%) of FoxP3^+^ expression in the SP and BM of 2-week-old mice. **(B)** Frequency (%) of FoxP3^+^ Treg in the SP and BM of aging mice from 2 to 52 weeks. **(C)** Representative histogram and% of TIGIT^+^ expression in 2-week-old mice **(D)** Frequency (%) of TIGIT^+^ Treg in the SP and BM of aging mice. **(E)** Representative histogram and frequency (%) of KLRG1^+^ expression in 2-week-old mice. **(F)** Frequency (%) of KLRG^+^ Treg in the SP and BM of aging mice. **(G)** gMFI of intracellular FoxP3 expression in TIGIT^neg^ and TIGIT^+^ CD4^+^CD3^+^ T cells isolated from the SP or BM of 2-week-old mice. **(H)** gMFI of intracellular FoxP3 expression in the SP and BM of aging mice. Data are shown as mean ± SEM, and statistical significance was determined using paired *t* test (*P < 0.05; **P < 0.01). Statistical analyses were performed using GraphPad Prism version 6.01 software.

At birth, Treg numbers in VAT are limited, but progressively accumulate over time with peak Treg numbers occurring at ∼25 weeks of age (Feuerer et al., 2009). To assess whether the BM-Treg, and particularly the VAT-like KLRG1^+^ Treg, exhibited a similar accumulation pattern, SP- and BM- Treg compartments were examined in mice aged from 2 weeks to 1 year old. At 2 weeks, there was already a significant FoxP3^+^ Treg enrichment in the BM CD4 T cell compartment (Figure 5A), which was maintained over time (Figure 5B). Similarly, in 2-week-old mice, TIGIT^+^ and KRLG1^+^ Treg subsets were significantly increased in BM-Treg compared to the SP (Figures 5C,E). The proportion of TIGIT^+^ Treg was consistently higher in the BM-Treg compartment; over time, the proportion of TIGIT^+^ Treg increased in both SP and BM (Figure 5D). In contrast, and opposed to Treg accumulation in VAT, the proportion of KLRG1^+^ BM-Treg reduced significantly after 2 weeks, and gradually declined thereafter (Figure 5F). In 2-week-old mice, Foxp3 expression was not reduced in BM-Treg as was seen for BM-Treg in older mice. Rather, we noted increased FoxP3 expression in the TIGIT^+^ BM-Treg (Figures 5G,H). Treg in neonates undergo robust expansion due to a lymphogenic environment (Min et al., 2003). Since proliferating Treg upregulate TIGIT expression, these data suggest that the reduced BM-Treg FoxP3 levels in adult mice may be related to their relative quiescent state.

BM-Treg have been reported as high producers of IL-10 which has direct effects on multiple populations within the BM (Fujisaki et al., 2011; Fischer et al., 2019; Camacho et al., 2020). We previously demonstrated an enrichment of IL-10 producing Treg within the TIGIT^+^ BM-Treg compartment (Le Texier et al., 2016; Yu et al., 2018). Using B6.FoxP3-RFPxIL10-GFP reporter mice, we identified IL-10^+^ Treg as a CD304^+^CD69^+^PD1^+^ memory Treg subset (Supplementary Figure 5). Notably, the KLRG1^+^ Treg subset lacked IL-10 expression. Although increased in proportion in the BM, the IL-10^+^ Treg phenotype was similar in both the SP and BM compartments, with the exception that IL-10^+^BM-Treg had a higher level of Sca1 expression. Together these data further support that BM harbors not only phenotypically, but functionally distinct subsets of Treg and that the BM microenvironment influences multiple aspects of BM-Treg phenotype and function.

### BM-Treg Exhibit Unique Cytokine Signaling Requirements

**FIGURE 6:**
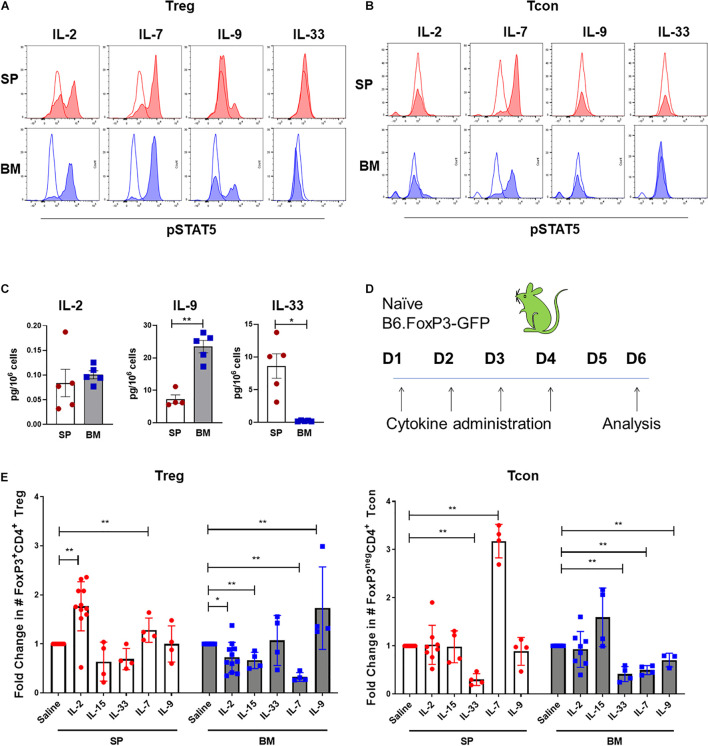
BM-Treg exhibit unique cytokine signaling requirements. **(A)** MFI of STAT5 phosphorylation (pSTAT5) in CD4^+^FoxP3^+^ Treg isolated from either the BM or SP following *in vitro* cytokine stimulation. **(B)** MFI of pSTAT5 in CD4^+^FoxP3^neg^ T cells isolated from either the BM or SP following *in vitro* cytokine stimulation. **(C)** Quantification of *in vivo* concentrations of IL-2, IL-9, and IL-33 in the BM and SP of naïve WT mice. **(D,E)** Naïve B6.FoxP3-GFP mice were administered with recombinant cytokines (IL-2, IL-7, IL-15, and IL-33) or saline for 4 days prior to analysis of Treg and Tcon populations in the SP and BM. **(D)** Schema of cytokine administration. **(E)** Fold change in the absolute number of FoxP3^+^ Treg and CD4^+^FoxP3^neg^ T cells in the BM and SP following cytokine administration. Data are shown as mean ± SEM, and statistical significance was determined using paired *t* test (*P < 0.05; **P < 0.01). Statistical analyses were performed using GraphPad Prism version 6.01 software. pSTAT5, STAT5 phosphorylation; WT, wild type.

Although IL-2 is critical for pTreg homeostasis, the quiescent status of BM-Treg correlated with a predicted reduction in IL-2 signaling consistent with the finding that IL-2mAb/IL-2 complexes failed to expand BM-Treg after transplant and suggesting an alternative cytokine(s) may function to maintain and shape the Treg in the BM niche. Since BM-Treg exhibited upregulated IL-9R, CD127 (IL-7R), and IL-33R (ST2) expression, we first compared phospho-STAT5 (pSTAT5) responses in BM and SP Treg following *in vitro* cytokine stimulation, and accordingly, observed increased pSTAT5 response in BM-Treg following *in vitro* IL-2, IL-7, and IL-9 stimulation (Figure 6A). IL-33 failed to elicit pSTAT5 in either population. Only IL-7 elicited pSTAT5 in FoxP3^neg^ Tcon that was similar in both BM and SP Tcon (Figure 6B). As the local cytokine milieu can influence cytokine receptor expression and cellular responses, and IL-7 is an established abundant cytokine in the BM, we assessed IL-2, IL-9, and IL-33 levels in the SP and BM niche. We found limited amounts of IL-2 in either tissue. IL-9 was significantly increased in the BM while IL-33 was more abundant in the SP than BM (Figure 6C), suggesting a potential role for IL-9 in modulating BM-Treg locally.

We next examined the impact of *in vivo* IL-7, IL-9, and IL-33 administration on Treg and Tcon populations is SP and BM. In addition to IL-2, IL-15 also acts as a potent inducer of pTreg mediated responses. Therefore, we compared responses to those elicited by IL-2mAb/IL-2 complexes or IL-15mAb/IL-5 complexes. Following 4 days of administration to naïve B6.FoxP3-GFP mice (Figure 6D), IL-2mAb/IL-2 complexes stimulated the greatest expansion of SP-Treg, with no expansion of Tcon. Similar to what we observed in cGVHD mice, there was little to no impact on BM-Treg numbers (Figure 6E). IL-15mAb/IL-15 complexes failed to increase SP-Treg numbers, however, BM-Treg were decreased and there was a statistical trend to increased Tcon in the BM. IL-33 had no impact on Treg in either the SP or BM, but decreased Tcon in both compartments. While IL-7 expanded both Tcon and Treg in the SP and is reduced in the BM. In contrast and in support of IL-9 as a BM-Treg support cytokine, IL-9 treatment significantly increased BM-Treg numbers in parallel with a decrease in BM Tcon in the BM but had no effect on splenic Treg or Tcon.

### pDCs Are Required for the Enrichment and Maintenance of BM-Treg

**FIGURE 7:**
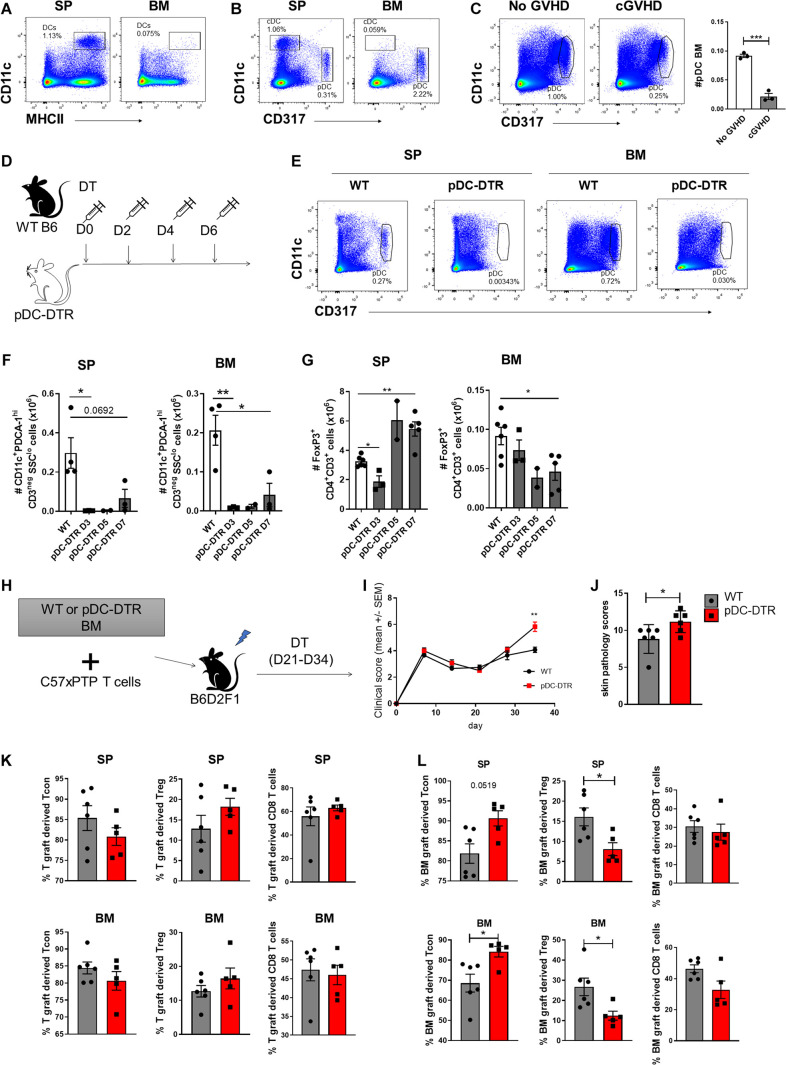
pDCs are required for the BM-Treg maintenance *in vivo*. **(A,B)** Flow cytometry analysis of dendritic cell populations in SP and BM of naïve C57BL/6 (B6) mice. **(A)** Representative frequency (%) cDC (CD11c^+^MHCII^+^) frequency (%) in the SP and BM. **(B)** Representative frequency (%) of pDC (CD11c^low^CD317^+^) in the SP and BM. **(C)** Irradiated allogeneic B6D2F1 (H2D^b/d^) recipients were transplant with B6 BM with (cGVHD) or without (No cGVHD) B6 SP T cells. Representative analysis and enumeration of pDC populations in the SP and BM WT mice following allogeneic SCT. **(D–G)** BDCA2-DTR transgenic mice (pDC-DTR) or naïve B6 mice were administered with DT for 1 week to illicit a global depletion of pDCs. **(D)** Outline of DT administration strategy. **(E)** Representative flow analysis of cDC and pDC populations in the SP and BM following DT administration. **(F)** Enumeration of pDCs in the BM and SP of pDC-DTR mice following DT administration. **(G)** Enumeration of CD4^+^FoxP3^+^ Treg in the BM and SP of pDC-DTR mice following DT administration. **(H–L)** Irradiated allogeneic B6D2F1 (H2D^b/d^) recipients were transplanted with either WT B6 or pDC-DTR BM, supplemented with B6 SP T cells to induce cGVHD. Transplanted mice were administered with DT twice weekly from day 21 until day 34 following transplant. T cell populations were quantified by flow cytometry on day 35 post-transplant. **(H)** Outline of allogeneic transplant strategy. **(I)** Clinical scores and **(J)** skin pathology scoring from histopathology analysis of B6 or pDC-DTR BMgraft recipients (*n* = 6). **(K)** Frequency (%) of Tgraft derived CD4^+^ Tcon and CD4^+^FoxP3^+^ Treg in the SP and BM of B6 (*n* = 6) or pDC-DTR (*n* = 5) BMgraft recipients. **(L)** Frequency (%) of BMgraft CD4^+^ Tcon and CD4^+^FoxP3^+^ Treg in the SP and BM of B6 (*n* = 6) or pDC-DTR (*n* = 5) BMgraft recipients. Data are shown as mean ± SEM. Statistical significance was determined using an unpaired 2-tailed Mann-Whitney U test or a paired *t* test where appropriate (*P < 0.05; **P < 0.01; ***P < 0.001). Statistical analyses were performed using GraphPad Prism version 6.01 software. cDC conventional dendritic cell; pDC, plasmacytoid dendritic cell; pDC-DTR, BDCA2-DTR transgenic mice.

Dendritic cells (DC) are required for the induction and maintenance of Treg both in steady state and after SCT (Stenger et al., 2012; Yu et al., 2019). Impaired function of conventional DC (cDC) and reconstitution of plasmacytoid DC (pDC) during GVHD have both been demonstrated as mechanisms underpinning failed Treg homeostasis (Banovic et al., 2009; Koyama et al., 2016; Tian et al., 2021). Comparison of the DC subset composition in the SP and BM of naïve mice demonstrated pDC (CD11c^dim^CD317^+^) as the predominant DC population, with limited contribution of CD11^hi^MHCII^+^ cDC in BM (Figures 7A,B). We confirmed impaired pDC reconstitution in the BM of mice with cGVHD in the B6 → B6D2F1 model (Figure 7C). We therefore reasoned that pDC, rather than cDC, may contribute to the local maintenance of Treg in the BM. To address this we used BDCA2-DTR transgenic mice (hereafter pDC-DTR) (Swiecki et al., 2010) to induce the specific depletion of pDC and examined the impact on Treg in the SP and BM. In naïve mice we confirmed rapid and maintained pDC depletion in SP and BM by DT administration (Figures 7D–F). On day 3 of pDC depletion, SP Treg numbers were reduced, but had rebounded by day 7. In contrast, BM-Treg showed a gradual and persistent reduction, supporting the notion that pDC preferentially contributed to BM-Treg maintenance (Figure 7G). SP Treg numbers increased to ∼ 2-fold over basal levels, indicative of niche filling activity (Pierson et al., 2013). In this regard, in the SP but not the BM, pDC depletion induced a marked increased Treg expression of the anti-apoptotic protein MCL-1, in parallel with a reduction in apoptosis, highlighting divergent homeostatic mechanisms in these tissues (Supplementary Figure 6).

To determine the impact of pDC depletion on the GVHD severity and Treg engraftment during cGVHD, we undertook transplants where BM from pDC-DTR or WT littermate donor mice were transferred with WT B6 T cells. pDC were deleted with DT administration from day 21 to 34 (Figure 7H). Following pDC depletion that was restricted to the BMgraft, we observed an increase in cGVHD severity and associated skin cGVHD pathology (Figures 7I,J). Enumeration of BMgraft and Tgraft Treg in the SP and BM on D35 after transplant revealed that while pDC depletion did not impact the frequency of Tgraft derived T cells (Figure 7K), we observed a significant reduction in BMgraft-derived Treg in both the BM and SP (Figure 7L). Taken together, these data demonstrate that pDCs within the BMgraft are critical for BM-Treg maintenance during GVHD. Thus, the absence of pDCs post-transplant further contributes to loss of Treg mediated control of GVHD leading in an increase in disease severity.

## Discussion

Regulatory T cell represent a diverse immunoregulatory regulatory population necessary for peripheral tolerance and immune homeostasis. This is particularly evident within the setting of allogeneic SCT, where Treg have been shown to be critical for immune reconstitution and GVHD control. There is a significant defect in the reconstitution and maintenance pTreg following SCT (Chen et al., 2007; Beres and Drobyski, 2013; Le Texier et al., 2016) that is directly implicated in the perpetuation and exacerbation of GVHD severity (Taylor et al., 2002; Rezvani et al., 2006; Robb et al., 2012; Zhang et al., 2013; Le Texier et al., 2017). Similarly, here we demonstrate that while the BM niche represents a rich source Treg following SCT, BM-Treg also fail to reconstitute during GVHD. We provide evidence that BMgraft-derived Treg play a functional role in controlling GVHD and show that restricted depletion of BM-Treg, while preserving splenic Treg, significantly increases disease severity in mice.

To combat GVHD in the clinical setting, several current strategies aim to globally increase pTreg frequency *in vivo* to reduce the severity and incidence of GVHD in SCT recipients. We have previously shown anti-IL-2 mAb/IL-2 complexes can drive a robust expansion of pTreg, thereby reducing GVHD severity (McDonald-Hyman et al., 2016). In this present study, we demonstrate that while anti-IL-2 mAb/IL-2 complexes can effectively expand splenic Treg, minimal impact on BM-Treg expansion during cGVHD is seen indicating that other approaches are needed to reestablish BM-Treg following SCT and improve GVHD control.

Previous studies have demonstrated that BM-Treg play a central role in supporting HSC engraftment and are important for BM homeostasis (Fujisaki et al., 2011; Hirata et al., 2018, 2019; Kakiuchi et al., 2021). However, many of the mechanisms that locally influence BM-Treg phenotype and function remain unclear. Here we expand upon previous phenotypic analyses and provide further evidence that BM-Treg represent a phenotypically heterogeneous and distinct Treg population. We demonstrate that cellular and soluble mediators within the local tissue microenvironment support BM-Treg maintenance, function, and reconstitution *in vivo*. Consistent with previous reports, we demonstrate that BM-Treg exhibit several signatures indicative of a highly functional, activated memory phenotype. In addition to observing elevated expression of memory and activation markers in BM-Treg (Le Texier et al., 2016; Camacho et al., 2020), including TIGIT, KLRG1, CD69, and CD127, our curated analysis also identified other Treg-associated gene transcripts that differed in BM-Treg. For example, the expression of Sca-1, an activation and memory marker associated with “boosted” Treg populations (i.e., stimulated by CD4^+^ or CD8^+^ Tcon) (Grinberg-Bleyer et al., 2010), was significantly upregulated in BM-Treg. Although there is some evidence to suggest that Sca-1 is not a restrictive marker of memory T cells (DeLong et al., 2018), high BM-Treg Sca-1 expression coupled with elevated KLRG1 and CD69 (Le Texier et al., 2016; Kornete et al., 2017; Yu et al., 2018; Camacho et al., 2020) is typically associated with a mature memory-like T cell phenotype. BM-Treg also exhibited highly elevated expression Fgl2, a well-established Treg immunosuppressive effector molecule that has previously been linked to TIGIT^+^ Treg subsets. In fact, Fgl2 expression in TIGIT^+^ Treg has been shown to be critical for Treg mediated control of T cell helper cells type-1 (Th1) and Th17, but not Th2, populations (Joller et al., 2014; Chruscinski et al., 2015).

Conversely, our analysis also suggests that BM-Treg are more quiescent and less functional compared to pTreg. The BM-Treg quiescent state correlated with the downregulated expression of several genes typically associated with optimal Treg-mediated suppression, including FoxP3, Ki67, and GARP and reflected by a reduction *in vitro* suppressive function compared to splenic Treg. Thus, the BM niche provides an environment that can maintain memory T cells in a quiescent state, retain features of their memory-like phenotype (Okhrimenko et al., 2014; Kalia et al., 2015; Di Rosa, 2016; Di Rosa and Gebhardt, 2016) and contributes to the maintenance of long-lived memory T cells for disease control and immune homeostasis (Di Rosa, 2016; Di Rosa and Gebhardt, 2016).

Our data suggest that the BM niche is acting as a biological resting site for pTreg by supporting the survival of long-lived memory Treg, while allowing them to retain distinct elements of their highly functional Treg signatures. In line with this observation, transcriptomics revealed that several Treg markers associated with Treg migration and homing were upregulated, notably, ITGA2 and CD103. The increased frequency of CD103^+^ Treg, specifically within the TIGIT^+^ subset is indicative of a highly activated and suppressive phenotype (Lehmann et al., 2002; Huehn et al., 2004). CD103 is also a well-established homing maker associated with T cell homing to sites of acute inflammation (Huehn et al., 2004); its receptor, E-cadherin, is also highly expressed in BM stroma cells, potentially accounting for the enrichment of BM-Treg (Turel and Rao, 1998; Morrison and Scadden, 2014). Similarly, ITGA2, which encodes CD49b, has been shown to be a marker of Treg type-1 (Tr1) and IL-10 producing Treg (Gagliani et al., 2013; Huang et al., 2018), and an established homing marker for T cell migration to the BM compartment (Tokoyoda et al., 2009). While the role of upregulated EpCam expression in BM-Treg is not yet defined, EpCam has been suggested to play a role in cell-to-cell adhesion (Linnenbach et al., 1989; Balzar et al., 1998; Schnell et al., 2013)(85-87). CD7, the ligand for EpCam, is highly expressed in pluripotent HSC (Laurson et al., 2005; Schnell et al., 2013)(85, 88), suggesting that HSC within the BM niche could also be directly contributing to the recruitment and retention of Treg. Additionally, we previously reported the BM-Treg express high levels of CXCR4 which mediates preferential migration of Treg to the BM niche (Zou et al., 2004; Sugiyama et al., 2006; Le Texier et al., 2016). Here, we expand upon this previous analysis and suggest that elevated expression of CD103, CD49b and EpCam in BM-Treg may also be integral in regulating Treg migration and retention in the BM niche. Through recruitment and/or the local influence of the microenvironment, the BM harbors distinct and heterogeneous subsets of Treg.

The heterogeneity of BM-Treg can be seen throughout our phenotypic analysis which identified an enrichment of multiple sub-populations within the BM niche that doesn’t cumulate in a singular Treg phenotype. Here, we identified several distinct Treg subsets that were all highly enriched within the BM-Treg population, including KLRG1^+^ VAT-like tissue Treg, TIGIT^neg^, TIGIT^+^, and IL-10 producing Treg subsets. BM-Treg have previously been shown to be high producers of IL-10 and are the principal source of IL-10 within the BM (Fischer et al., 2019; Camacho et al., 2020). While IL-10 production is well-defined as potent immunosuppressive mechanism important for regulating inflammatory immune responses (Kornete et al., 2012; Schmidt et al., 2012; Ng et al., 2013), recent studies have also shown IL-10 plays a central role in mediating stromal cell function and hematopoiesis within the BM (Camacho et al., 2020). Our data suggests that IL-10 producing Treg are limited to several subsets of BM-Treg. We further demonstrate an enrichment of IL-10 producing Treg within the TIGIT^+^ Treg subset (Le Texier et al., 2016) within a CD304^+^CD69^+^PD1^+^ memory Treg phenotype. In contrast, increased expression of CD62L, KLRG1, and CD103 did not coincide with an elevated production of IL-10 from BM-Treg, again demonstrating that BM-Treg represent a heterogenous Treg population. Accordingly, such heterogeneity within the BM-Treg population spurs significant diversity in Treg function, likely the result of the accumulation and retention of multiple Treg populations from the periphery and local regulation of these Treg populations by the BM microenvironment, necessary for BM-Treg expansion, activation, and maintenance.

By using a lymphocyte-deficient Rag1^–/–^ mouse model we were able to demonstrate that adoptively transferred splenic Treg become enriched within the BM niche where, despite originating from a singular source, they adopt *in vivo* the distinct BM-Treg signature observed *in vitro*. There is growing evidence that Treg within different tissue niches throughout the body represent phenotypically and functionally distinct Treg subsets that locally maintain immune homeostasis in a tissue specific manner (Burzyn et al., 2013a; Zhou et al., 2015). The distinct Treg heterogeneity (Sakaguchi et al., 2010; Zhou et al., 2015) may be influenced by the immense diversity of cellular and non-cellular mediators in each specialized tissue environment. The BM represents a complex and highly specialized tissue niche (Babyn et al., 1998; Travlos, 2006; Romaniuk et al., 2016). In fact, it has been more recently suggested that BM tissue could be further sub-divided into functional niches in which the variable distribution of different elements that contribute to the composition of the BM, including HSC, stomal cells, adipose tissue, T cells, and soluble factors (i.e., cytokines), interact and differentially influence HSC and T cell functions such as cell proliferation, differentiation, migration, and quiescence (Shafat et al., 2017). As such, a better understanding of the tissue specific mechanisms that drive BM-Treg function *in vivo* is critical to improve current approaches to enhance Treg reconstitution and reestablish tolerance following SCT.

Bearing this in mind, we investigated several elements within the BM microenvironment that appeared to have a differential effect on BM-Treg and identified the upregulation of several cytokine and chemokine receptors in BM-Treg. Among these, BM-Treg had upregulated IL-9R expression and an enhanced capacity to respond to IL-9 both *in vitro* and *in vivo*. Importantly, we also observed significantly increased IL-9 levels within the BM niche, suggesting that BM-Treg readily adapt to the local cytokine milieu to preferentially respond to prevalent cytokines within the tissue niche. As IL-9 signaling is required for optimal maintenance of Treg suppressor function (Karagiannis and Wilhelm, 2017; Rauber et al., 2017)(98, 99), an enhanced response to IL-9 signaling pathways may provide a distinct survival and functional advantage for BM-Treg. This is markedly different from the response to IL-2 stimulation which, despite driving robust expansion of splenic Treg, had little to no effect on BM-Treg expansion. The reduced response to IL-2 in BM-Treg may be the result of dysregulated IL-2 mediated signaling pathways, as our analysis noted reduced expression of GARP in BM-Treg. GARP, also known as the TGFβ-R1 (Wang et al., 2009; Zhang et al., 2015), can be driven by IL-2 signaling (Zhou et al., 2013). As Ingenuity IPA predicted IL-2 to be the third most significantly downregulated canonical pathway in BM-Treg, BM-Treg may be intrinsically less capable of responding to IL-2 than splenic Treg. Despite high IL-7Ra (CD127) expression that has also been noted as a distinguishing feature of BM-Treg (Simonetta et al., 2010; Camacho et al., 2020), we demonstrate that *in vivo* IL-7 administration does not impart a selective advantage toward Treg expansion within the BM niche. Rather, the opposite was seen, as pTreg and Tcon populations were more capable of expansion in response to IL-7 than BM-Treg. However, as BM-Treg have a robust response to *in vitro* IL-7 stimulation, it is also possible that IL-7 signaling induces BM-Treg activation, expansion and simultaneously instigating release of activated BM-Treg into circulation. In aggregate, these data demonstrate that BM-Treg have vastly different and adaptive cytokine signaling requirements compared to other Treg and Tcon populations.

Among other cytokine receptors upregulated in BM-Treg, ST2 expression was highly elevated, even though IL-33 stimulation did not illicit a pSTAT5 response in BM-Treg. Rather, ST2 expression marked a distinct BM-Treg subset that co-expressed KLRG1, indicative VAT-like Treg signature (Hu and Zhao, 2015). TCF-1 expression was significantly downregulated in BM-Treg; a hallmark of non-lymphoid tissue Treg, including those accumulated within VAT (Wang and Fu, 2020). Previous work identified PPARγ as the major driving force behind the development of the VAT Treg phenotype (Cipolletta et al., 2012; Wang and Fu, 2020) and we find that PPARγ is upregulated in BM-Treg. As fatty acids from adipose tissue are naturally occurring ligands of PPARγ (Wagenmakers et al., 2006; Tontonoz and Spiegelman, 2008), we propose the natural enrichment of adipose tissue within the BM niche may be a significant driving force in the development of this VAT-like BM-Treg subset. In contrast to Treg accumulation in peripheral VAT (Feuerer et al., 2009), the frequency of KLRG1^+^ Treg only encompasses a small proportion of BM-Treg and declines in aging mice, suggesting that the BM-Treg signature may readily adapt to local shifts within the BM microenvironment over time. Adipose tissue accounts for up to 70% of the total BM volume in healthy adults (Blebea et al., 2007; Cawthorn et al., 2014, 2016; Li et al., 2019). Unlike peripheral adipose tissue which develops in distinct deposits, adipose tissue in the BM develops unevenly with concentrations of fatty tissue in the distal tibia and caudal vertebrae (Scheller et al., 2015; Hardouin et al., 2016; Sebo et al., 2019) that could be further contributing to the distinct heterogeneity of BM-Treg.

Similar to the notable influence of adipose tissue on the regulation of BM-Treg, recent studies have identified several complex interactions between BM-Treg and their local microenvironment which directly contribute to the function and homeostasis of the BM niche itself (Fujisaki et al., 2011; Fischer et al., 2019; Hirata et al., 2019; Camacho et al., 2020; Kakiuchi et al., 2021). Notably, previous findings have suggested that BM-Treg are essential for HSC survival and engraftment post-transplant, as they are capable of establishing an immune privileged site within the BM niche (Fujisaki et al., 2011; Hirata et al., 2018), and have also been shown to protect HSCs from oxidative stress by regulating quiescence within the BM niche (Hirata et al., 2018, 2019). Additionally, BM-Treg have been identified to support the function of mesenchymal stromal cells (MSCs), and thereby support hematopoiesis, via IL-10 secretion *in vivo* (Camacho et al., 2020). Interestingly, MSCs have also been known to enhance the immunosuppressive function of immune cells, including Treg populations (Lee et al., 2017; Khosravi et al., 2018; Zhang et al., 2018). These studies provide additional evidence of extensive cellular cross talk between BM-Treg and their local tissue microenvironment. However, the scope of many of these studies was focused primarily on the role of BM-Treg in the maintenance and function of BM microenvironment. We speculate that these interactions are likely bidirectional, and that other cell populations within the BM niche may also play integral roles in the regulation and maintenance of BM-Treg *in vivo*.

In support of this, we discovered a significant relationship between pDCs and BM-Treg *in vivo*. Previously, DCs have been shown to play an integral role in maintaining immune tolerance through the induction and activation of pTreg (Stenger et al., 2012; Yu et al., 2019). In particular, pDCs have drawn recent interest as a potentially tolerogenic DC population that has vastly different functional properties depending on their local tissue environment (Banovic et al., 2009; Thomson and Knolle, 2010; Cardenas et al., 2011). Consistent with previous literature (Banovic et al., 2009; Cardenas et al., 2011), pDCs are markedly enriched within the BM compartment at a steady state. A global pDC depletion in mice at a steady state correlates with a significant reduction in BM-Treg frequency. These data suggest that pDCs may play a direct role in maintaining the BM-Treg population but are not required for the maintenance of splenic Treg populations. Interestingly, our studies also show that pDCs, like BM-Treg frequencies, are significantly depleted within the BM niche during cGVHD, and recent studies have suggested that pDCs may be critical for control of GVHD severity (Reddy et al., 2004; Mohty et al., 2005; Vakkila et al., 2005). Having established that pDCs are required for the steady-state maintenance of BM-Treg, we proposed that BM resident pDCs are critical for the maintenance of BM-Treg during GVHD. A selective pDC depletion in the BMgraft of cGVHD mice had no effect on splenic T cell populations even in mice with a significant reduction in BMgraft-derived Treg, which correlated with a local and peripheral expansion of BMgraft-derived Tcon, indicating a loss of Treg mediated control within the BMgraft. Together, our data indicate that BM resident pDCs are critical for the maintenance of BM-Treg both at steady state and during GVHD. As such, the reduced frequency of pDCs following SCT is likely a key contributor to loss of BM-Treg mediated control of GVHD, reinforcing the idea of an extensive and complex crosstalk between environmental mediators within the BM niche and BM-Treg.

In conclusion, our study provides valuable insight into several complex signaling pathways that differentially regulate BM-Treg survival, expansion, and function *in vivo*. Due to the distinct phenotypic and functional differences between peripheral and BM-Treg, new, more tailored approaches are needed to reestablish and maintain BM-Treg populations and restore immune tolerance following SCT. Additional studies are needed to further delineate the direct and indirect mechanisms that regulate this diverse population.

## Data Availability Statement

The data presented in the study are deposited in the GEO repository, accession number GSE182502.

## Ethics Statement

Experiments were approved by and performed in accordance with the QIMR Berghofer Animal Ethics Committee and by the University of Minnesota Institutional Animal Care and Use Committee.

## Author Contributions

KM, BB, SN, BC, SP, and JN conceived and designed the experiments. JN, BC, LL, LX, CH, GL, EA, WS, JL, HC, SH, BW, AC, CN, JP, SN, and KM performed the experiments. JN, BC, LL, LX, CH, EA, WS, CN, SN, and KM analyzed and interpreted the data. KM, BB, SN, and SP contributed reagents and materials. JN, KM, and BB wrote the manuscript. All authors contributed to the article and approved the submitted version.

## Conflict of Interest

The authors declare that the research was conducted in the absence of any commercial or financial relationships that could be construed as a potential conflict of interest.

## Publisher’s Note

All claims expressed in this article are solely those of the authors and do not necessarily represent those of their affiliated organizations, or those of the publisher, the editors and the reviewers. Any product that may be evaluated in this article, or claim that may be made by its manufacturer, is not guaranteed or endorsed by the publisher.
