# Supplementary material for: Bone Marrow Regulatory T Cells Are a Unique Population, Supported by Niche-Specific Cytokines and Plasmacytoid Dendritic Cells, and Required for Chronic Graft-Versus-Host Disease Control
Source: Front Cell Dev Biol. 2021 Sep 22;9:737880. doi: 10.3389/fcell.2021.737880 (PMC8493124; doi:10.3389/fcell.2021.737880)
Supplement: Supplementary file 1 [file Data_Sheet_1.PDF]

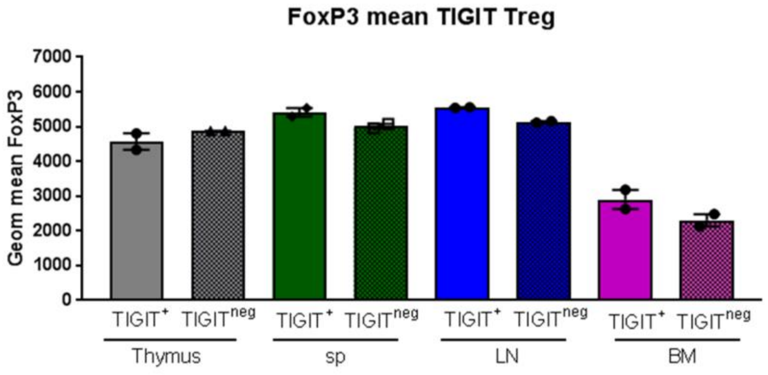

Supplemental Figure 1: BM-Treg have reduced expression of FoxP3 compared to Treg in other lymphoid organs. Geometric mean (gMFI) of intracellular FoxP3 expression in $TIGIT^{neg}$ and $TIGIT^{+}$ $CD4^{+}CD3^{+}$ T cells isolated from the Thymus, SP, lymph nodes (LN) or BM on naïve WT B6 mice (*n* = 2). Treg, regulatory T cell; TIGIT, T cell immunoreceptor with Ig and ITIM domains; SP, spleen; BM, bone marrow; LN, lymph nodes; WT, wild type.

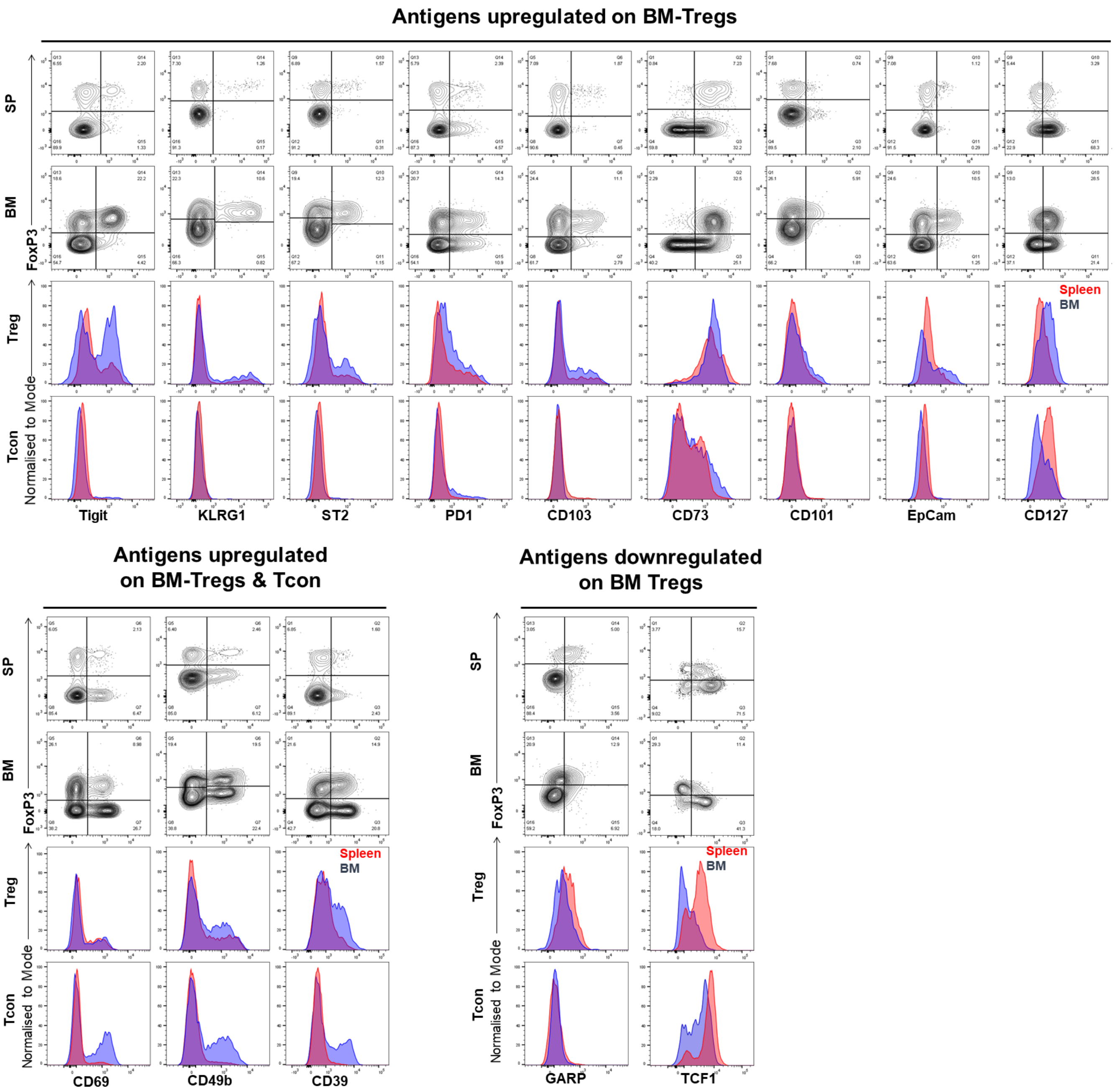

Supplemental Figure 2: Differential expression of functional markers in BM- and SP-Treg. Flow cytometry analysis of differential expression of functional markers on CD3^{+}CD4^{+}FoxP3^{neg} Tcon and CD3^{+}CD4^{+} FoxP3^{+} Treg from the SP and BM of 12-week-old WT C57BL/6 mice. Data is shown as representative contour plots illustrating functional marker expression relative to FoxP3 expression, and representative gMFI histograms for each marker in FoxP3^{neg} Tcon or FoxP3^{+} Treg in the SP and BM.

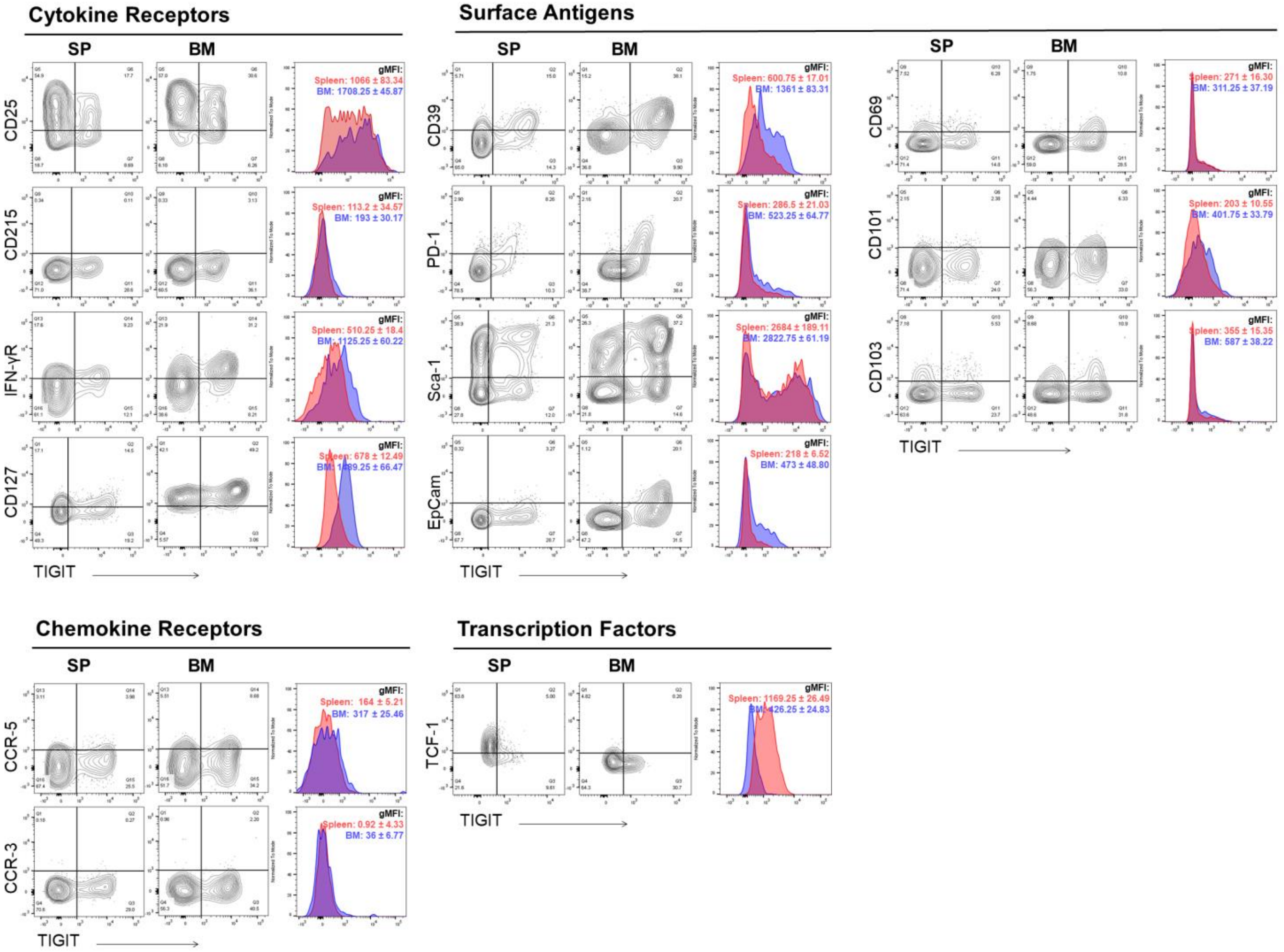

Supplemental Figure 3: Relative expression of Treg functional markers relative to TIGIT expression in BM-Treg. Flow cytometry analysis of Treg associated functional markers and the relative expression in TIGIT$^+$ and TIGIT^{neg} CD4$^+$FoxP3-GFP$^+$ Treg sub-populations from the SP and BM of 12-week-old B6.FoxP3-GFP reporter mice (*n* = 4). Data is shown as representative contour plots illustrating functional marker expression in FoxP3$^+$ Treg relative to TIGIT expression in the SP and BM, with representative histogram overlays and gMFI (±SEM).

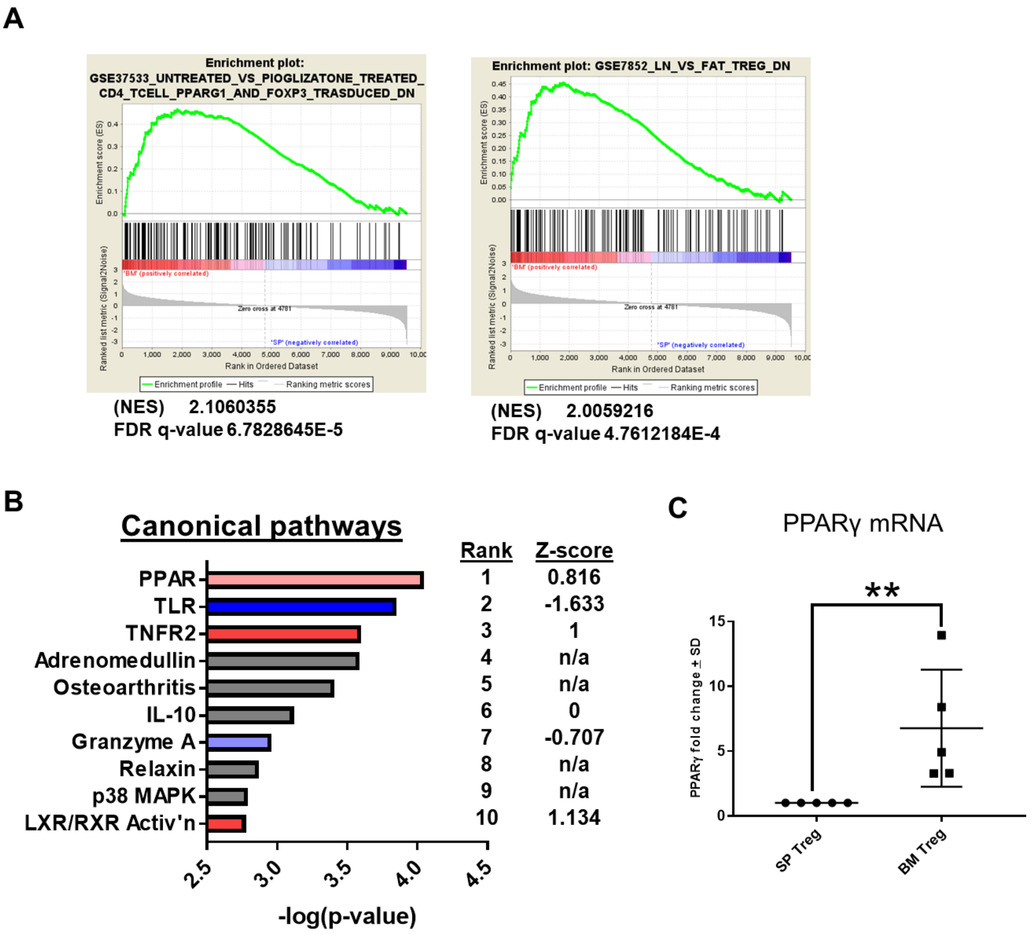

Supplemental Figure 4: PPARγ signaling pathways are upregulated in BM-Treg. (**A**) GSE analysis of bulk RNAseq comparing BM-Treg and SP-Treg against two previously publish data sets (56, 57). (**B**) Ingenuity IPA of likely dysregulated USRs in BM-Treg compared to SP-Treg. (**C**) RT-qPCR analysis of PPARγ mRNA expression in BM-Treg and SP-Treg (n = 5). Data are shown as mean ± SEM, and statistical significance was determined using paired t test (*$P < 0.05$; **$P < 0.01$). Statistical analyses were performed using GraphPad Prism version 6.01 software. GSE, Gene Set Enrichment; RNAseq, RNA sequencing; SP, spleen; BM, bone marrow; IPA, interpretative phenomenological analysis; USRs, upstream regulators; RT-qPCR, real time quantitative PCR; mRNA, messenger RNA.

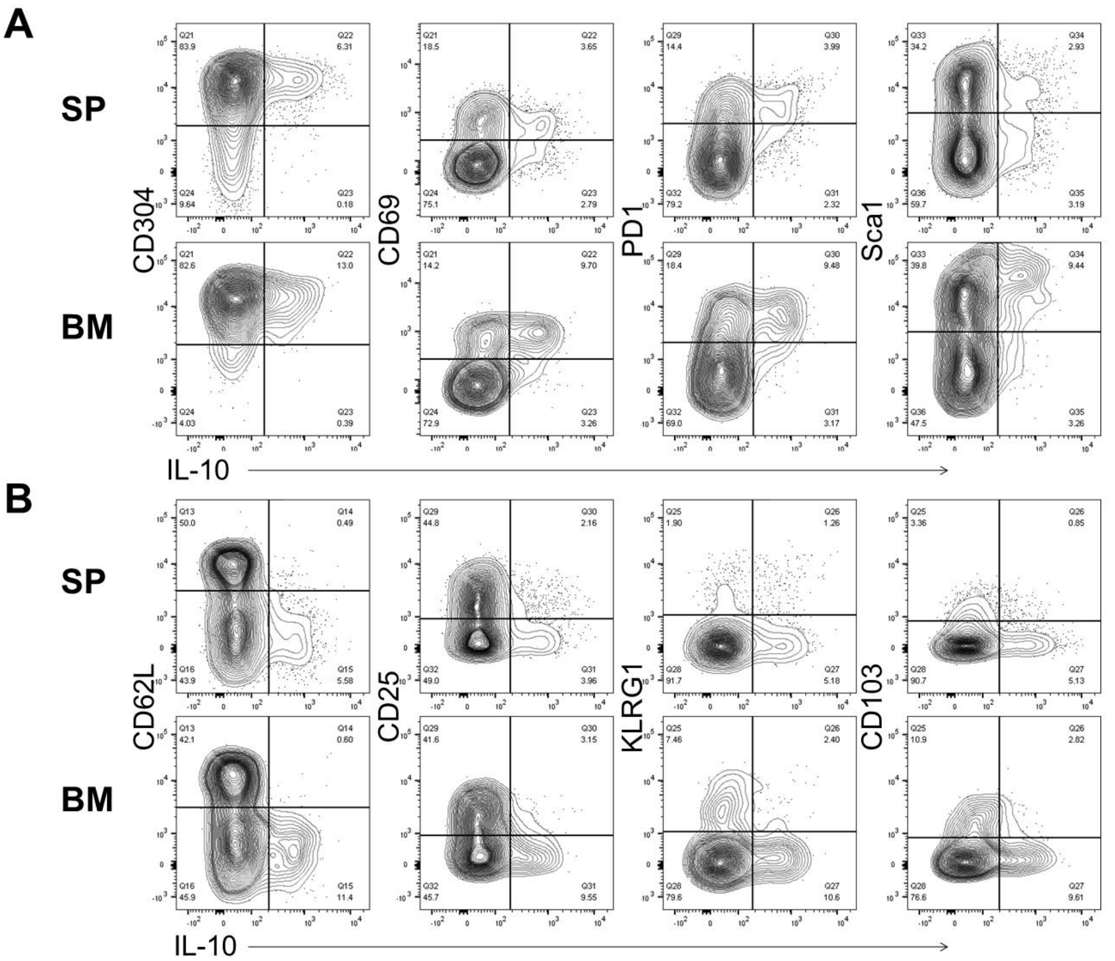

Supplemental Figure 5: Enrichment of IL-10$^+$ Treg within the BM niche is restricted to a memory Treg subset. Flow cytometry analysis of IL-10$^+$ Treg populations in the SP and BM of 12-week-old B6.FoxP3-RFPxIL10-GFP reporter mice. (**A**) Treg subsets corresponding with an enrichment in IL-10$^+$ expression. (**B**) Treg subsets that are not shown to co-express IL-10.

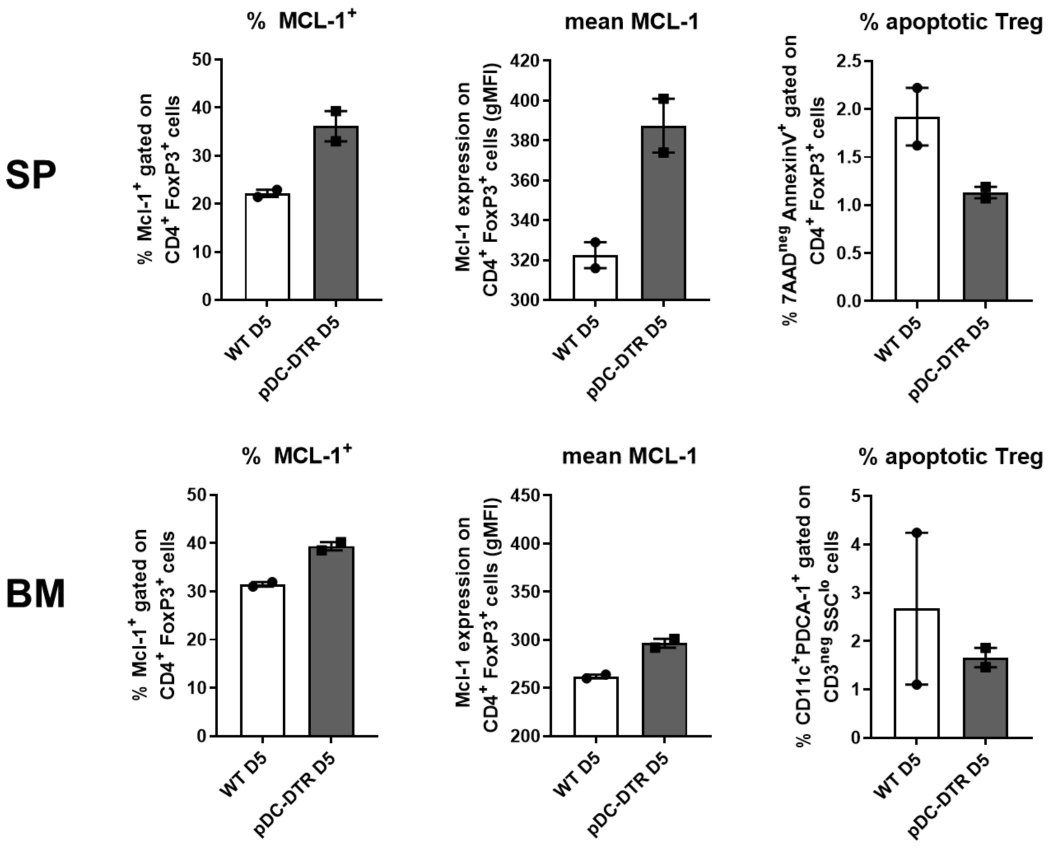

Supplemental Figure 6: pDC depletion in naïve mice causes marked increases in MCL-1 expression and reduced apoptosis in SP-Treg and not BM-Treg at steady state. pDC-DTR or naïve WT B6 mice were administered with DT to elicit a global depletion of pDCs. Frequency (%) and gMFI of MCL-1 expression in Treg isolated from the SP or BM of WT or pDC-DTR mice following DT administration. Frequency (%) of AnnexinV^{+} Treg in SP and BM of WT or pDC-DTR mice following DT administration. Data are shown as mean ± SEM. Treg, regulatory T cell; pDC, plasmacytoid dendritic cell; pDC-DTR, BDCA2-DTR transgenic mice; WT, wild type; DT, diptheria toxin; SP, spleen; BM, bone marrow.
